# A Distance-Based Framework for the Characterization of Metabolic Heterogeneity in Large Sets of Genome-Scale Metabolic Models

**DOI:** 10.1016/j.patter.2020.100080

**Published:** 2020-08-06

**Authors:** Andrea Cabbia, Peter A.J. Hilbers, Natal A.W. van Riel

**Affiliations:** 1 Computational Biology, Eindhoven University of Technology, Groene Loper 5, 5612 AE Eindhoven, the Netherlands; 2 Amsterdam University Medical Centers, University of Amsterdam, Meibergdreef 9, 1105 AZ Amsterdam, the Netherlands

**Keywords:** metabolism, genome-scale metabolic models, heterogeneity, machine learning, distance

## Abstract

Gene expression and protein abundance data of cells or tissues belonging to healthy and diseased individuals can be integrated and mapped onto genome-scale metabolic networks to produce patient-derived models. As the number of available and newly developed genome-scale metabolic models increases, new methods are needed to objectively analyze large sets of models and to identify the determinants of metabolic heterogeneity. We developed a distance-based workflow that combines consensus machine learning and metabolic modeling techniques and used it to apply pattern recognition algorithms to collections of genome-scale metabolic models, both microbial and human. Model composition, network topology and flux distribution provide complementary aspects of metabolic heterogeneity in patient-specific genome-scale models of skeletal muscle. Using consensus clustering analysis we identified the metabolic processes involved in the individual responses to endurance training in older adults.

## Highlights

• Distance metrics express heterogeneity between genome-scale metabolic models
• Flux distribution and network topology are the main components of metabolic distance
• Machine learning algorithms are applicable to sets of genome-scale metabolic models
• We model individual responses to endurance training in older adults

## The Bigger Picture

High-throughput techniques enable the analysis of complex biological systems at multiple levels, including genome, transcriptome, proteome, and metabolome. Integration of multi-omics data is often focused on dimensionality reduction and feature selection for classification tasks. Genome-scale metabolic models are extensive maps of the network of biochemical reactions taking place in a particular cell, tissue or organism. Each reaction is associated with the respective enzyme and gene, enabling the mapping of transcriptomics and proteomics data and providing a structure for the system-level interpretation of multi-omics datasets. The result of this process is a personalized model that gives a snapshot of the metabolic status of an individual. Analyzing these complex models, for example, to detect differences between individuals, is cumbersome. We applied consensus clustering to a set of data-driven models to monitor the progression of a lifestyle intervention in a cohort of older adults.

## Teaser

Genome-scale metabolic models are maps of the metabolic network that function as structures for the integration of molecular data, such as transcriptomics and proteomics. We developed a method for the analysis of large sets of data-driven models, using different distance metrics to quantify model similarity. Consensus analysis is then used to reach a single metabolic distance. The method was applied to model the individual variability in the responses to endurance training in a cohort of older adults.

## Introduction

Individual differences in genetic backgrounds and lifelong exposure to different environmental stressors are responsible for the variability in the outcomes of medical interventions within a population. Methods to identify clinically relevant subgroups of individuals who respond similarly to the same treatment and estimate their future outcome for a given treatment would be relevant both in research and for clinical practice, for example, to study the heterogeneous pace of aging observed in different individuals,^1^^,^^2^ or to develop targeted interventions against the development of frailty with age.^3^ Current methods for patient stratification and prediction of health outcomes are broadly divided in data driven and physiology driven. Data-driven methods seek statistical correlations between the outcome and one or more covariates, while physiology-based (dynamic) models use previous biological knowledge, structured in differential equations, to explain experimental data. Nevertheless, both approaches have certain disadvantages: statistical correlations are insufficient to explain the mechanism of a disease, while physiology-based dynamic models have a limited scope and scale since they are difficult to parametrize.

Genome-scale metabolic models (GSMMs) take a third, alternative approach: they mathematically reproduce the network of metabolic reactions happening inside an organism or tissue and can be used to simulate the response to specific environmental conditions, such as the availability of nutrients. The activity of each reaction in the model can be linked to experimental data, such as levels of gene expression and protein abundance, making such models a combination of data-driven and physiology-driven methods and interesting platforms for the system-level integration of multi-omics datasets.^4^, ^5^, ^6^ Advancements in automated model generation algorithms^4^^,^^7^^,^^8^ have made possible the construction of large collections of GSMMs derived from experimental data, such as cancer patient-derived models,^9^ and microbial models of the human gut microbiota.^10^^,^^11^ Despite this increase in the number of new models generated and published, few studies so far have tackled the issue of heterogeneity across GSMMs.^12^^,^^13^ The current approach to describe the heterogeneity in a model set is to apply a similarity metric:^10^ used the Jaccard distance to describe the heterogeneity in a collection of GSMMs of gut microbiota, and the same metric has been used to describe the differences between the output of alternative model reconstruction algorithms.^14^^,^^15^ Other metrics, such as Hamming distance^16^ and Pearson's correlation coefficient^12^ have been used as alternatives to describe heterogeneity within model sets. In this study, we argue that distance metrics are also key to the application of supervised and unsupervised pattern recognition methods to genome-scale model sets.

Machine learning (ML) algorithms are able to identify patterns in very large datasets, relying on distance metrics to quantify similarity between different data points.^17^, ^18^, ^19^ GSMMs parametrized with experimental data can be considered as a novel data class that comprises both information about the structure of the metabolic network and multiple types of omics data, upon which ML algorithms could be applied.^20^^,^^21^ The detection of patterns contained in large model collections would help determine the mechanisms behind the heterogeneity in individual responses to metabolic and pharmacological interventions.

We identified several different similarity metrics for model composition, network topology, and flux distribution and compared their properties by applying them to two large GSMMs sets: the AGORA set,^10^ composed of 818 models of human gut microbiota, and a subset of the PD-GSMM set,^9^ composed of 100 human tissue-specific cancer metabolic models. We developed a distance-based workflow that, by combining ML and metabolic modeling, was able to identify biologically relevant patterns present in a novel set of 24 GSMMs of skeletal muscle metabolism in older adults. These models were developed from longitudinal gene expression data that were gathered from the muscle tissue of 12 healthy older adults, before and after completing a 12-week resistance training program. Two models were created for each individual: one representing the baseline condition or “untrained” (UT) status, the other representing the “after training” (AT) status. The three metrics provide complementary aspects of metabolic heterogeneity in patient-specific genome-scale models. Using consensus clustering analysis we identify metabolic processes involved in the individual responses to endurance training in older adults.

## Results

### Distance Metrics Enables Consensus Clustering and Visualization of GSMM Sets

GSMMs are complex data structures with three main features: they are databases of metabolic genes and the associated metabolites and biochemical reactions which were experimentally found or predicted to be present in a certain tissues or organisms; they are mathematical representations of the topology of the metabolic network; and they are used to simulate how the metabolism of a tissue or organism adapts to a particular environment or condition by limiting with upper and lower bounds the metabolic fluxes through exchange reactions with the external medium.

Starting from the idea of Euclidean distance between data points as a similarity measure in ML, we asked how this concept could be expanded and applied to GSMMs. In this context, distance can be thought of as a proxy for functional similarities between the metabolisms of two organisms. As an example, let us consider Bacilli and Clostridia, two families of sporulating bacteria belonging to the same phylum (Firmicutes). What distinguishes them is the fact that the former is aerobic, and the latter is not.

**Figure 1:**
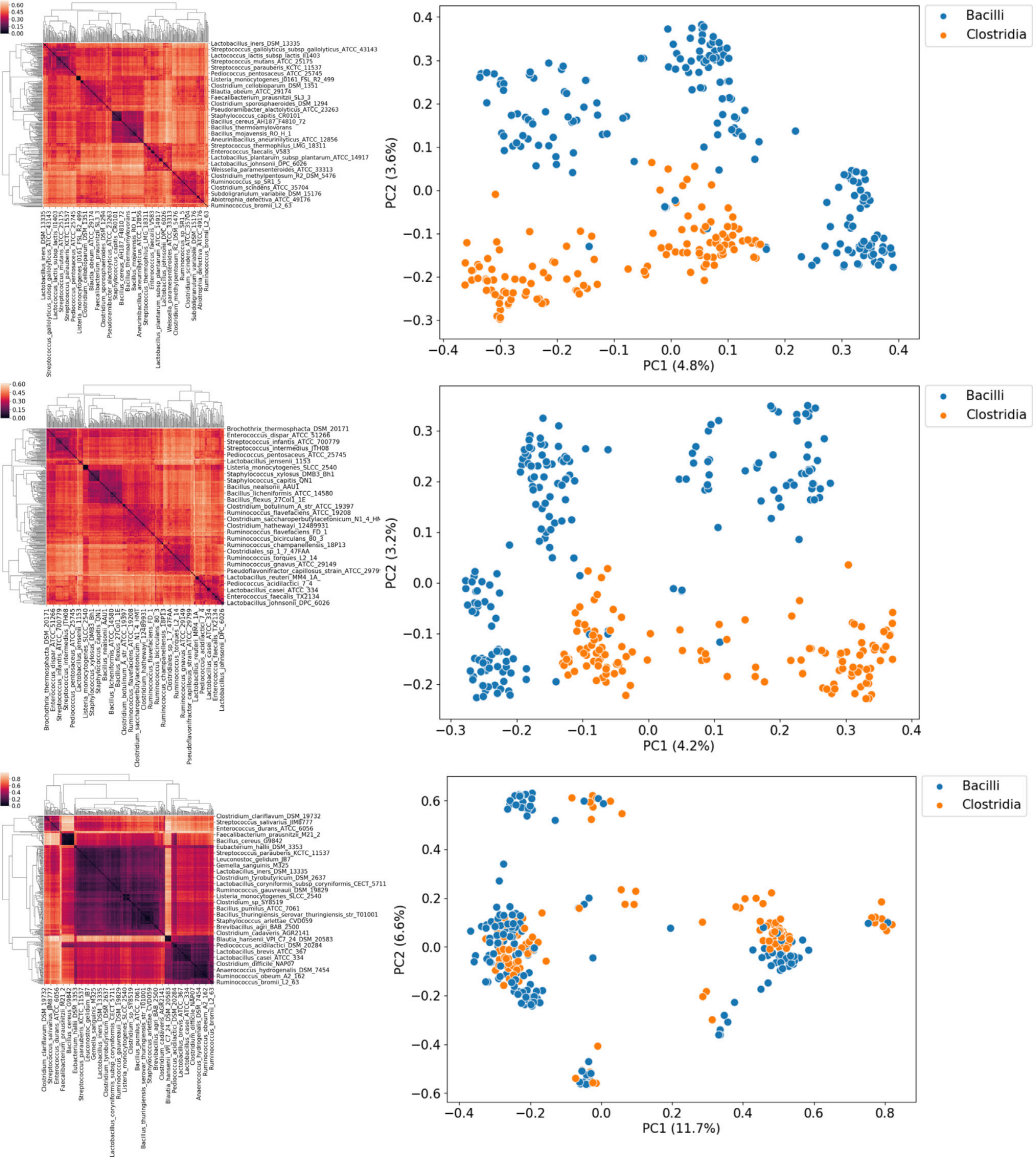
Distance Metrics Enable Consensus Clustering Analysis and Visualization of GSMM Sets Comparison of three distance metrics applied to a subset of the AGORA model set (Bacilli and Clostridia, n = 328 models). From top to bottom: Jaccard distance, Weisfeiler-Lehman subtree (WLS) kernel distance, flux distribution correlation. Left: heatmaps of the three distance matrices that were used as input in for the consensus clustering algorithm. Right: results of the embedding of the same distance matrices, obtained via kernel PCA. Variance of each principal component is reported in parentheses.

**Figure 2:**
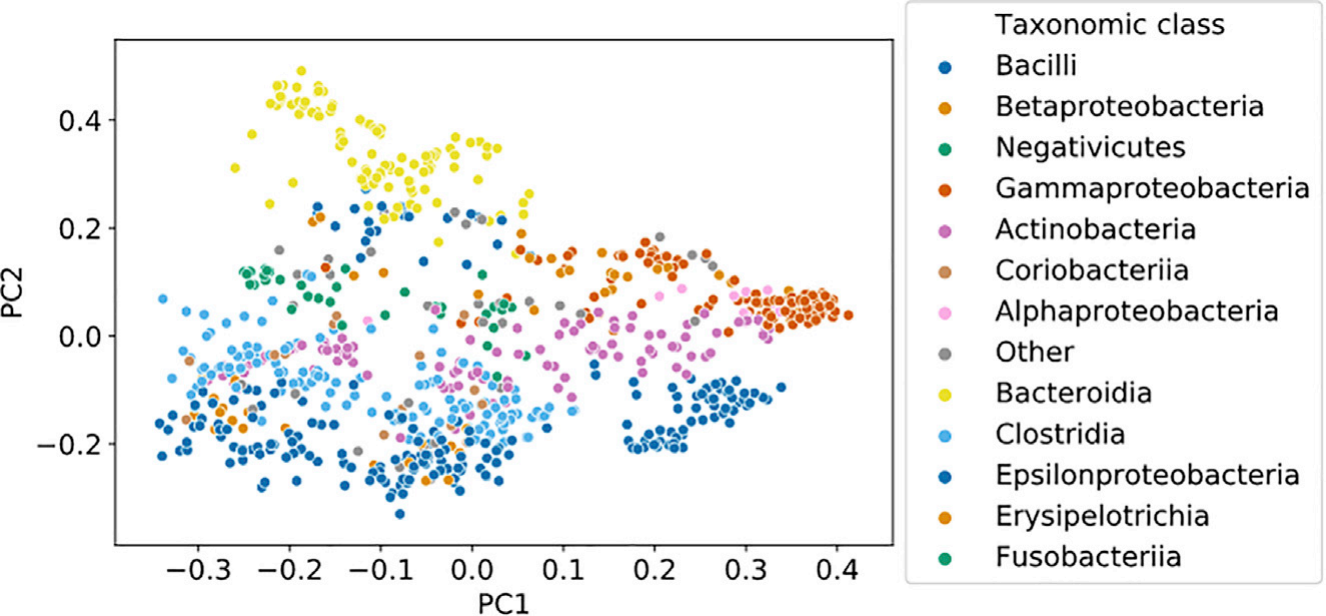
Visualization of the Full AGORA Microbial Model Set kernel PCA embedding of the AGORA dataset (n = 817 models), obtained using Jaccard distance. Variance of each principal component is reported in parentheses.

One way to quantify their similarity is by measuring the proportion of biochemical reactions they have in common. This information is summarized by the Jaccard metric. We computed the pairwise Jaccard distance between hundreds of bacterial models belonging to the Bacilli (n = 197) and Clostridia (n = 131) families, which were included in the AGORA model set. The results are visualized in Figure 1D. The two bacterial families are clearly distinguishable on the basis of the proportion of their shared reactions (i.e., their Jaccard distance), implying that distance is correlated with functional similarity. Another example is presented in Figure 2, which is a visualization of the Jaccard distance between each model in the AGORA model set: here we can observe how models belonging to the same taxonomic family cluster close to each other. The Jaccard metric is the *de facto* standard to measure similarity between genome-scale models.^10^^,^^14^^,^^15^

**Figure 3:**
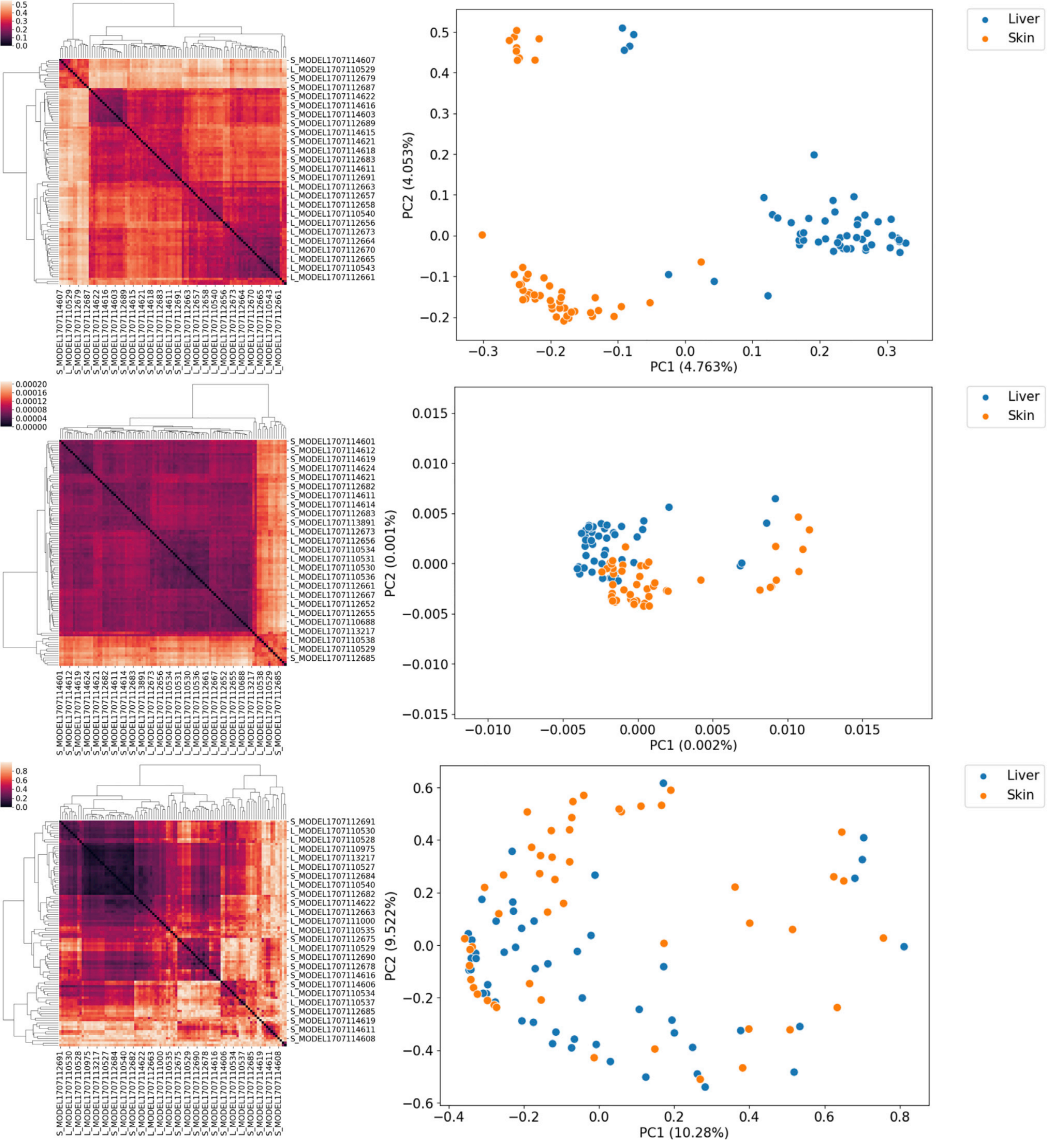
Comparison of the Three Metrics for a Subset of the PD-GSMM Set (Liver and Skin Cancer Models, n = 100) From top to bottom: Jaccard distance, WLS kernel distance, flux distribution correlation. Left: heatmaps of the three distance matrices that were used as input in for the consensus clustering algorithm. Right: results of the embedding of the same distance matrices, obtained via kernel PCA. Variance of each principal component is reported in parentheses.

We hypothesized that the heterogeneity within each of these three complementary components contributed to the “total” model heterogeneity and defined a different distance metric for each of them: the Jaccard metric was used to compute the overlap between the reaction lists of a pair of models; A graph kernel, i.e., a function to compute similarity between network topologies, was used to obtain a similarity score between metabolic networks. Pearson's correlation coefficient was instead used as similarity score between normalized flux distribution vectors as a metric of similarity between constraint-based models. The flux distributions are obtained averaging 1,000 random samples of the solution space of each model, then the flux through each reaction is normalized by subtracting the mean and dividing by the standard deviation of each reaction. A more detailed discussion of these metrics can be found in the Experimental Procedures section. To compare the properties of these metrics, we computed the pairwise distances for the models in the AGORA and PD-GSM datasets for each of the three different distance metrics. To test our hypothesis, we then looked at the correlation between the resulting pairwise symmetric distance matrices. The clustered distance matrices for each of the three metrics are shown in Figures 1A–1C and 3A–3C, respectively, for the AGORA and the PD-GSMM sets. Kernel PCA, a non-linear variant of principal-component analysis,^22^ was used to embed and visualize the pairwise distance matrices, allowing the visual inspection of the clustering results and of the overarching structure of the model set.

### Reaction List Similarity and Network Similarity Are Highly Correlated

Figure 1 illustrates how the Jaccard (Figure 1D) and graph kernel (Figure 1E) metrics can give similar representations of this model collection, and how in both cases the two classes are well separated. In the third case, flux correlation (Figure 1F), we can instead observe three clusters, a main one containing elements of both classes, and two smaller one containing only elements of one class. Contrary to our initial expectations, Jaccard distance and graph kernel distance showed an almost perfect correlation (Table 1), suggesting that these two metrics actually convey much of the same information. Jaccard distance does not only measure similarity between the reaction content of two metabolic reconstructions, but also expresses similarity between their network topologies. The third metric, Pearson's correlation between flux distributions, shows only a modest correlation with reaction content and network topology. This observation suggests that flux distribution and network topology/composition are the two complementary aspects of metabolic heterogeneity.

**Table 1:** Reaction List Similarity and Network Similarity Are Highly Correlated

	Jaccard	Graph Kernel	Flux Correlation
Jaccard	1.000	0.986	0.197
Graph kernel	0.986	1.000	0.189
Flux correlation	0.197	0.189	1.000

Pearson's correlation coefficients between pairs of distance matrices computed with different metrics for the full AGORA dataset (n = 817 models) obtained via the Mantel test. p values < 0.001.

The modest correlation between network structure, described by the first two metrics, and the flux correlation metric (Table 1) can be explained by the degeneracy of the metabolic network, i.e the existence of many possible flux distributions for a given topology. While the topology sets the limits of the possible states of the network, infinite different flux distributions are possible given the same topology. The composition and topology of the metabolic network is inferred from the genome of an organism or tissue: for example, metagenomic data from gut microbiota were used to draft the metabolic networks of the bacteria in the AGORA dataset.^10^ For any given environment, an organism will attain only one particular metabolic flux distribution, in principle the one that “optimizes” their fitness. This cellular behavior can be simulated with optimization techniques, such as flux balance analysis (FBA), which identifies the flux distribution that maximizes a certain cellular objective, for example, biomass production, when the organism is subjected to certain nutritional constraints. This particular solution can be thought of as the “metabolic phenotype” expressed by that organism under that particular condition.^23^

### Flux Distribution and Network Composition Are the Two Main Aspects of Metabolic Heterogeneity

To test if the observations we made regarding distance between microbial models are valid also for human metabolic models, we studied the distances in a subset of the PD-GSMM set, containing 100 patient-derived models of skin and liver metabolism.

Figure 3 shows how different metrics can give vastly different representations of the same model set. In the case of the Jaccard metric (Figure 3D), the two classes are well separated, forming two main clusters and two smaller ones. The graph kernel representation (Figure 3E) gives a different picture, showing instead two tight and overlapping clusters. In the third case, flux correlation (Figure 3F), the models are widely spread, but the two classes are even more overlapped.

The correlation between the metrics (Table 2) is lower than in the AGORA model set; nevertheless the Jaccard and graph kernel distance matrices still remain very highly correlated. Given the larger size of the human metabolic network compared with bacterial ones, the graph similarity metric may not have enough resolution to discriminate between the two classes. The difference between the networks in the PD-GSM set are too small, relative to their size. Looking at these results we can already conclude that our initial hypothesis, that metabolic heterogeneity can be described by three different metrics, is not supported by the data: the very high correlation between Jaccard and graph kernel shows that Jaccard is able to summarize the similarity of the metabolic networks as well as the overlap in the reaction content of two models.

**Table 2:** Flux Distribution and Network Structure Are Two Complementary Aspects of Metabolic Heterogeneity

	Jaccard	Graph Kernel	Flux Correlation
Jaccard	1.000	0.892	0.127
Graph kernel	0.892	1.000	0.151
Flux correlation	0.127	0.151	1.000

Pearson's correlation coefficients between pairs of distance matrices computed with different metrics for the PD-GSM dataset (n = 100 models) obtained via the Mantel test. p values < 0.001.

Given the low correlation between flux correlation and the other two metrics, flux distribution similarity should be viewed as a separate and complementary component of metabolic heterogeneity. Nevertheless, when we applied this metric to the PD-GSMM set, the two classes overlapped significantly. From an ML perspective, the flux correlation metric could not discriminate between the two classes. A classifier algorithm trained using this similarity metric would have worse performance than one trained using the Jaccard metric. For these reasons we decided to use only the Jaccard metric as a measure of metabolic heterogeneity for the remainder of this study.

### Description of the Proposed Workflow

**Figure 4:**
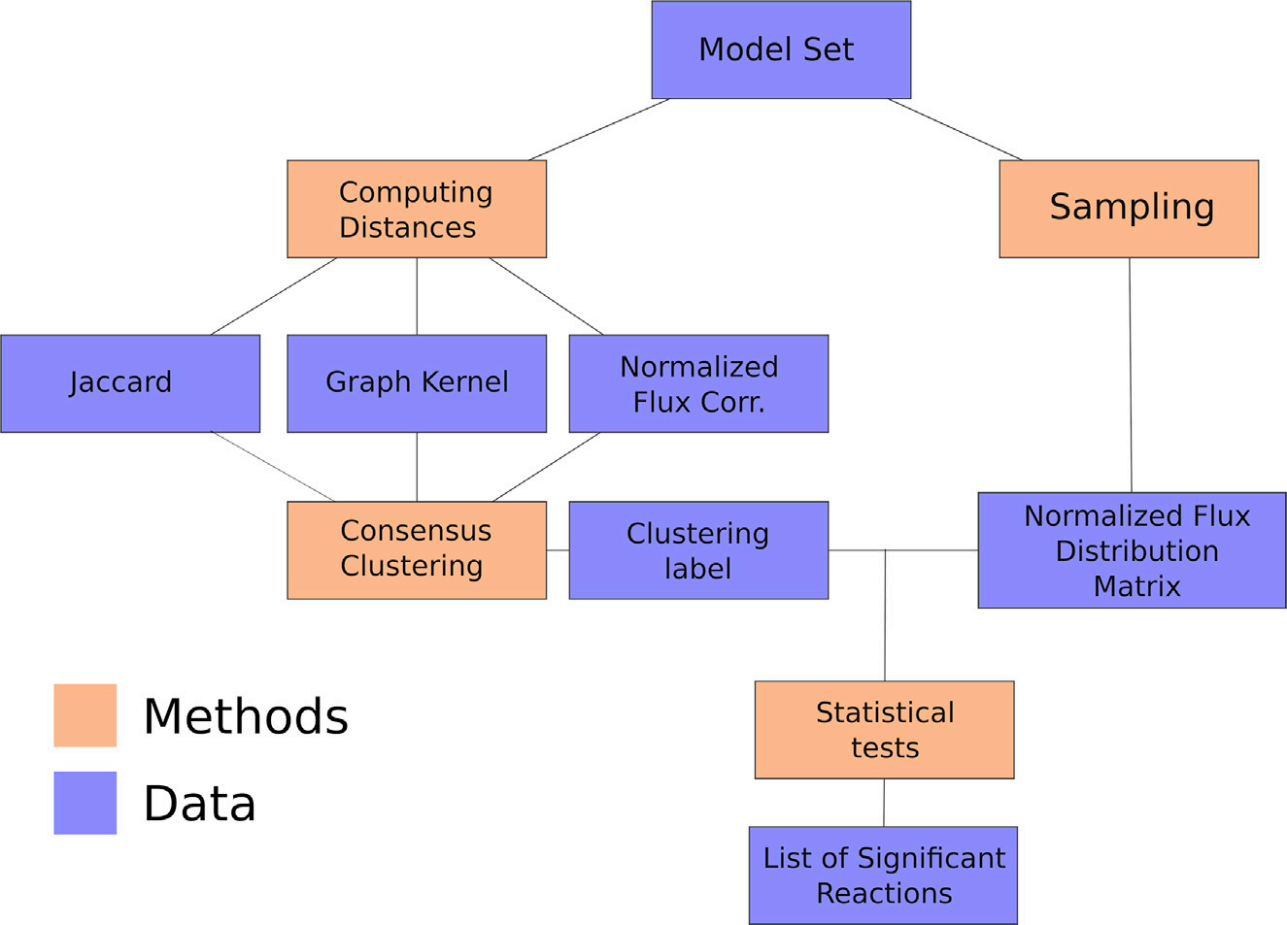
Description of the Proposed Workflow Three metrics of distance are computed between each pair of models. The resulting square distance matrices are used as input for the consensus clustering algorithm. The consensus label and a matrix of normalized flux distributions obtained via random sampling are used in the Kruskal-Wallis test to identify reactions whose activity differs between the clusters.

The publication of large GSMM collections, such as AGORA for gut microbiota models and PD-GSM for individualized human cancer models, showed the need for new scalable methods of analysis that can be applied to large model sets. We were interested in the concept of distance between individualized models and whether it could be applied to obtain biologically relevant insights about a set of GSMMs. Once we established the properties of distance metrics in GSMMs, we developed a distance-based workflow for the characterization of heterogeneity in metabolic models sets that, through the combination of ML and constraint-based metabolic modeling techniques, such as FBA,^24^ enables the visualization of the large-scale structure of the model set, allows cluster analysis of the models and identifies the set of reactions that differentiates the clusters. The workflow is presented in Figure 4.

The distance between each pair of models is computed using three different metrics: Jaccard distance, graph kernel and flux correlation, resulting in three square distance matrices of size n×n, where *n* is the number of models in the set. Each of the three metrics gives a different view of the heterogeneity present in a given model set. A consensus clustering approach is used to reconcile these differences and give a more comprehensive and robust view of metabolic heterogeneity: agglomerative hierarchical clustering is applied separately to each of the three distance matrices, then a consensus algorithm is applied to find an agreement between the three label vectors. For this task, we considered three consensus algorithms: the cluster-based similarity partitioning algorithm, the hyper graph partitioning algorithm, and the meta-clustering algorithm.^25^ In each of these algorithms, the optimization problem is reformulated in terms of a (hyper) graph partitioning problem. Among the three solutions, the definitive consensus is identified as the one with the highest average mutual information score between the input label vectors and the output consensus label vector. To visualize the structure of the model set, the pairwise distance matrices were transformed into a set of coordinates in an abstract Cartesian space using kernel PCA, an embedding algorithm. The output of this embedding can alternatively be used as input for other non-kernel-based ML algorithms.

The label predicted by the consensus clustering algorithm is used to investigate the differences in the flux distributions of the models between the clusters. A flux distribution matrix is created, merging the flux distribution vectors obtained via averaging 1,000 random samples of the solution space of the models. The flux matrix is then normalized, by subtracting the mean for each flux and dividing by its standard deviation. Using the Kruskal-Wallis test,^26^ a non-parametric version of ANOVA, is possible to identify the reactions whose activity differs between the clusters.

### Development of Patient-Derived GSMMs of Aging Skeletal Muscle Metabolism Before and After Metabolic Intervention

Skeletal muscle plays an important role during the aging process, not only in maintaining the ability to perform activities of daily living, but also because it increases resilience against stressors and adverse events, such as traumatic injuries and diseases.^27^ Low muscle mass and strength are predictors of mortality and adverse health outcomes in older adults,^28^ and are associated with development of frailty, a multifactorial condition of disability and increased vulnerability to adverse external stimuli.

Nutritional and physical activity interventions are currently the only possible treatments to slow muscle wasting in older adults. Nevertheless, aging individuals express a large variation of health outcomes, due to causes ranging from genetic differences to socioeconomic and behavioral factors,^29^ and not all individuals will experience the same magnitude of benefit from a given treatment.^30^ We are interested in modeling the heterogeneity of the individual responses to interventions that could improve health in older adults, from a metabolic point of view. Modeling the distance between GSMMs derived from individual data could help find the mechanisms behind the difference in individual responses to the same metabolic intervention and help the development of metabolic markers for each intervention.

**Figure 5:**
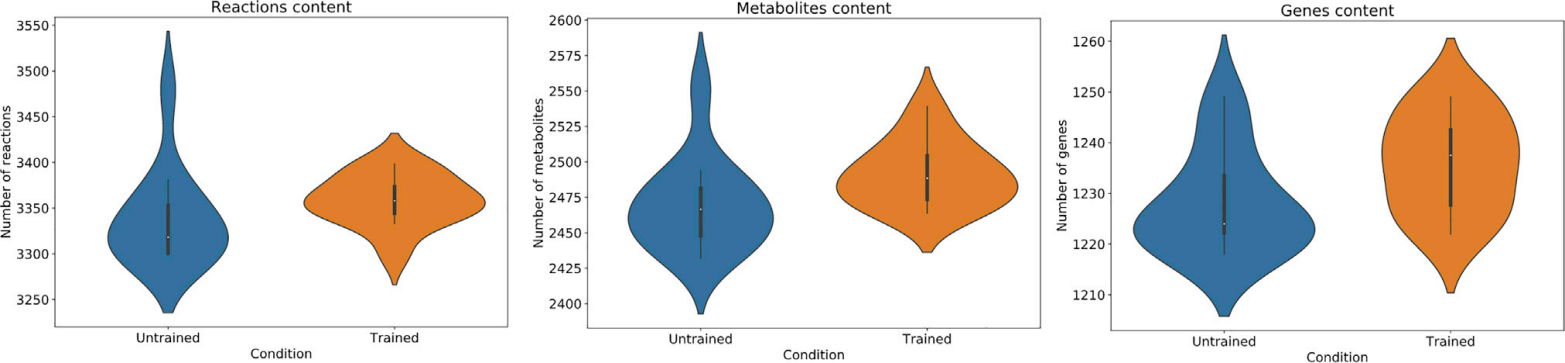
Distribution of the Contents of the Models in the Skeletal Muscle Model Sets From left to right: reaction content for the models of the untrained and trained classes; genes content distributions for the two classes; metabolites content distributions for the two classes. n = 12.

We developed an original set of 24 patient-derived GSMMs from longitudinal gene expression data of skeletal muscle of 12 older adults (84 ± 1 years old), before and after an intervention consisting of 12 weeks of resistance training. Although previous studies have investigated the metabolism of skeletal muscle using GSMMs,^31^^,^^32^ none has used longitudinal data to develop pairs of PD-GSMM and examine the response to a metabolic intervention, such as resistance training in a group of older individuals. All the models are based on a general human metabolic reconstruction, Recon2.2.^33^ Gene expression data were used to identify the reactions with experimental support, while a model generation algorithm, CORDA,^8^ was used to create drafts of the models, keeping only high-confidence reactions and a minimal number of lower-confidence reactions to ensure the functionality of the resulting network. Details about the model-building procedure are presented in the Experimental Procedures section. Information about the size and content of the resulting models for each of the two conditions are presented in Figure 5 and Table 3.

**Table 3:** Overview of the Content of the Models in the Skeletal Muscle Model Set

	Untrained	Trained
Median no. of reactions	3,318	3,358
Median no. of metabolites	2,466	2,488
Median no. of genes	1,224	1,237

Median number of reactions, metabolites and genes for the two classes of models in the skeletal muscle model set.

Figure 5 describes the content of the models included in the aging skeletal muscle model set in terms of reactions, genes, and metabolites for each of the two classes. UT models contain on average 42% of the reactions, 46% of the metabolites, and 73% of the genes of the “parent” model, Recon2.2, while AT models contain on average 44% of the reactions, 47% of the metabolites and 79% of the genes of the parent model.

The observation that AT models are on average somewhat larger than UT models (Table 3), suggests that individualized genome-scale models are able to capture at least in part the increased baseline gene expression after the end of the training program that was reported in the original study.^34^

### Clustering and Visualization of the Skeletal Muscle Model Set

**Figure 6:**
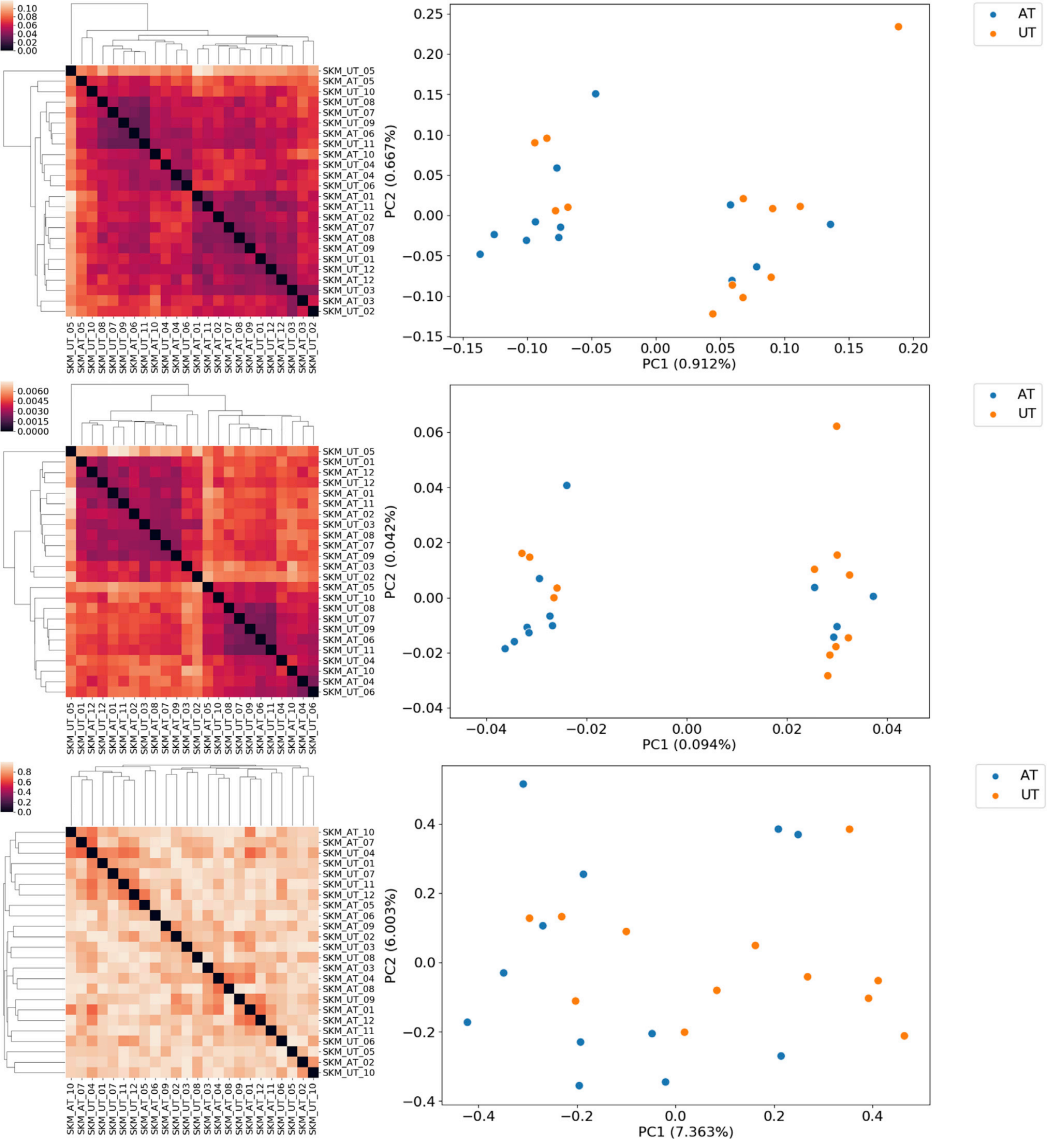
Comparison of the Three Metrics on the Skeletal Muscle Model Set (n = 24) From top to bottom: Jaccard distance, WLS kernel distance, flux distribution correlation. Left: heatmaps of the three distance matrices that were used as input in for the consensus clustering algorithm. Right: results of the embedding of the same distance matrices, obtained via kernel PCA. Variance of each principal component is reported in parentheses.

We applied our distance-based workflow to visualize the relationships between the models in the skeletal muscle model set and to perform a cluster analysis. The first step was to compute the Jaccard distance between each pair of models. The pairwise distance matrix was clustered with hierarchical clustering, and presented as a heatmap in Figure 6A. Figure 6B presents the embedding of the distance matrix, obtained via kernel-PCA. While it is immediately possible to identify two well-separated clusters, the two classes are not perfectly segregated as each of the clusters contain elements of both classes. The two clusters are enriched respectively in trained (left cluster) and UT (right cluster) models. One model, UT no. 5, is detected as an outlier.

### Metabolic Interventions as Trajectories in the Metabolic Health Space

Health is not a single immutable state but rather can be defined as a set of dynamic processes at different points in time that together form a health trajectory.^35^ The ability to model the health trajectories of different individuals over time to understand the causes of different individual responses to the same interventions and how the present condition affects future health would have a large impact not only on basic biomedical research, but also in clinical practice.

**Figure 7:**
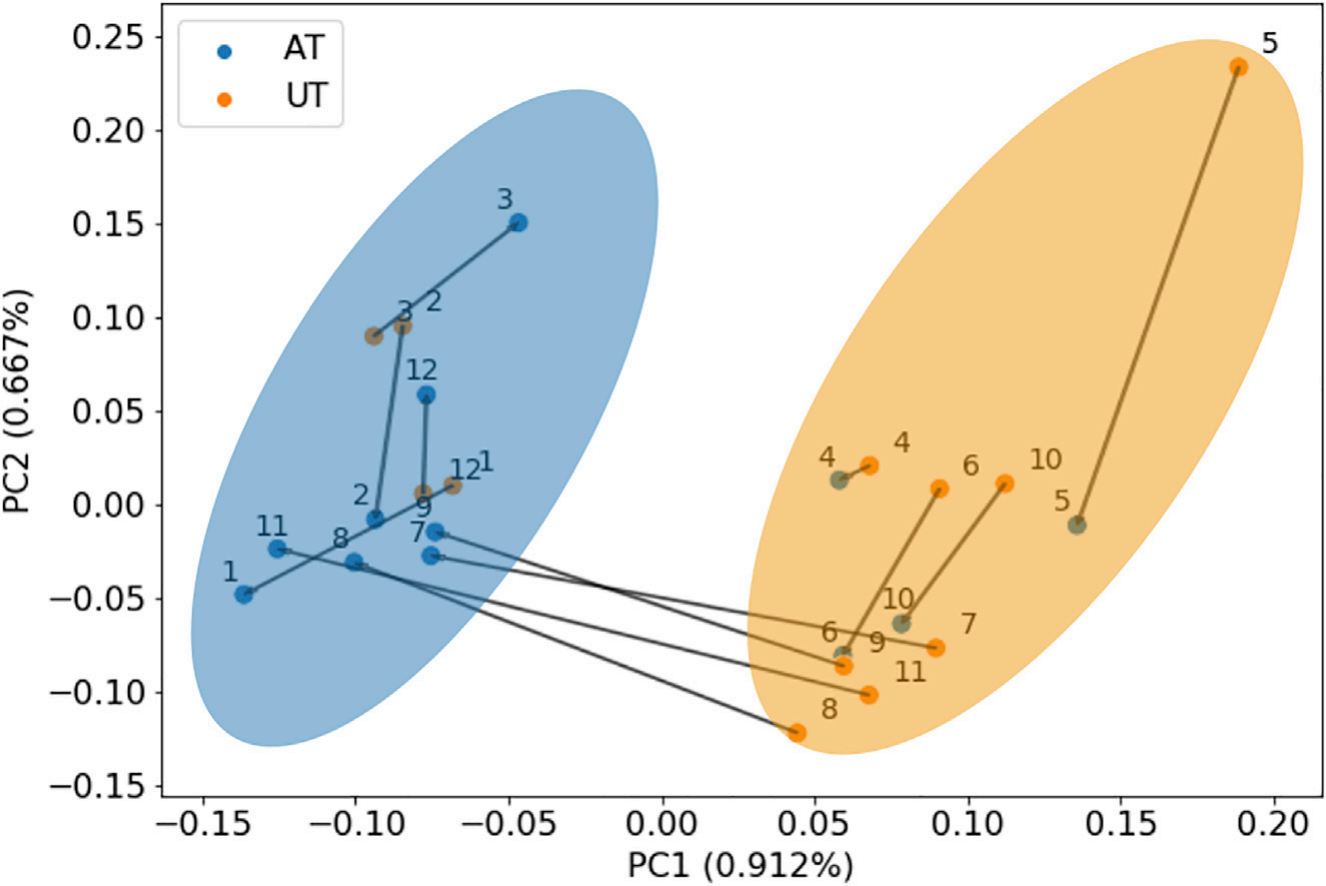
Kernel-PCA plot showing the metabolic trajectories taken by the individuals during the 12-weeks training program From top to bottom: Jaccard distance (A and D), WLS kernel distance (B–E), flux distribution correlation (C–F). Left: heatmaps of the three distance matrices that were used as input in for the consensus clustering algorithm. Right: results of the embedding of the same distance matrices, obtained via kernel PCA. Variance of each principal component is reported in parentheses.

The ability to model the health trajectories of different individuals over time to understand the differences in their responses to the same interventions and how their present condition affects future health would have a large impact not only on basic biomedical research, but also in clinical practice. Previous examples include modeling the progression of biological treatment in a “health space,” whose axis represent biological processes, such as oxidative stress, inflammation, and metabolism.^36^ The availability of pre- and post-intervention gene expression data allowed us to model the tissue-specific metabolic response of each individual to a lifestyle-change intervention (Figure 7). By tracing the arrows that join the two training states for each of the individuals in the cohort, it was possible to visualize the metabolic response to training in older adults as a trajectory in the metabolic health space.

The different directions and magnitudes of these shifts in the metabolic space reveal the heterogeneity of the individual response to the training program. The results of consensus clustering are visualized as colored shapes. Three main responses can be observed: individuals 4, 6, and 10 show a limited shift and remain in cluster 1 (orange) with mostly UT models even after the training is finished. UT model no. 5 starts as an outlier, but clusters together with UT models at the end of the intervention and was included in this group. Individuals 1, 2, 3, and 12 remain in the blue cluster, which includes mostly AT individuals, but also show a limited shift during the training. Conversely, individuals 7, 8, 9, and 11, who were starting from the orange cluster on the right, show the largest shift during the training intervention, clustering with other AT models in the blue cluster after the end of the treatment.

Since this study is based on publicly available data, we do not have any information about the physiological status or lifestyle of these individuals (e.g., frailty score, muscle strength, muscle mass, nutritional status, and diet), neither at baseline nor in response to the intervention. Thus we can only speculate whether the three different responses that we detected are correlated with the baseline fitness of the individual and to actual improvements in their condition after the intervention. For this reason, we will compare the models both according to the ground truth (i.e., AT versus UT) and to the predicted classes (i.e., cluster 0 versus cluster 1), inspecting the reaction content and average flux distribution of the models in each of the two groups.

### Detection of Condition-Specific Patterns from a Set of GSMMs

In the last step of the workflow, we use a statistical test (Kruskal-Wallis) to analyze the differences between flux distributions across groups of models and identify the metabolic processes associated with a certain class of models. We applied this step both to the ground truth label (i.e., AT versus UT models) and to the label predicted by the consensus clustering (i.e., cluster 0 versus cluster 1).

The Kruskal-Wallis test is applied to a normalized flux distribution matrix obtained by random sampling of the solution space of each model in the set. All the exchange reactions of the models were constrained with input flux values that reproduce the nutritional intake of an average European diet (retrieved from www.vmh.life/#nutrition). The statistical test is used to screen for reactions whose activity differs between the groups, using a p value of 0.05 as threshold. In total, we identified 147 reactions whose activity significantly differed between AT and UT models, and 238 significant reactions when comparing models of consensus clusters 0 and 1. The full list of significant reactions and associated p values, for both cases, is available in Tables S1 and S2.

**Figure 8:**
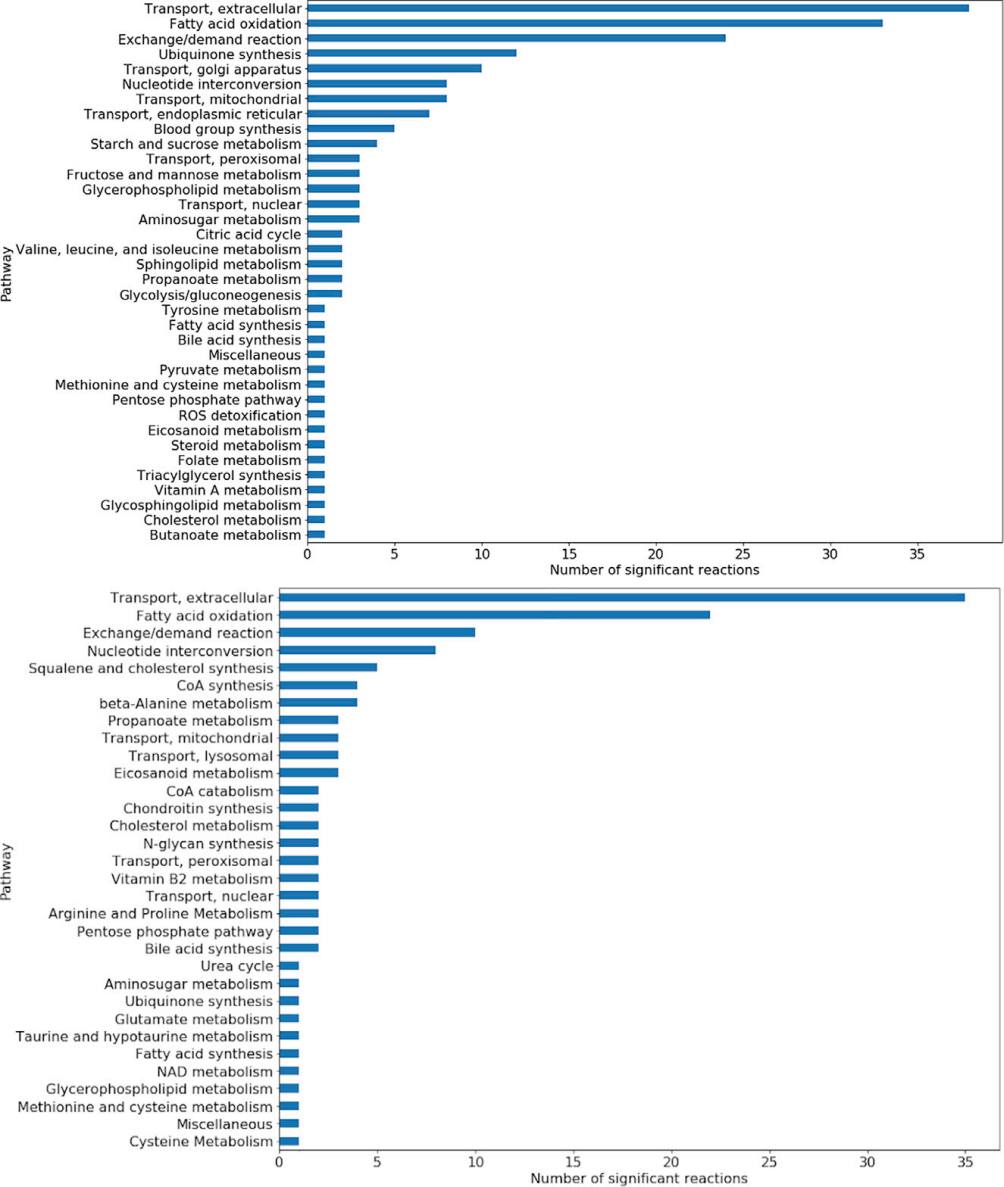
Number of Significant Reactions Identified for Each Pathway Top: number of significant reactions per pathway(p<0.05), AT versus UT models. Bottom: number of significant reactions per pathway, cluster 0 versus cluster 1 models

The most represented pathways associated with resistance training, except for transport and exchange reactions, were fatty acid oxidation, nucleotide interconversion, tryptophan metabolism, cholesterol/squalene metabolism, and beta-alanine metabolism (Figure 8, top panel). Inspecting the exchange reactions, i.e., the reaction responsible for exchange of metabolites from the simulated culture medium into the extracellular space, we can have an idea of how metabolic demands change in consequence of the intervention. Most of the selected reactions were related to exchange of fatty acids (linolenic acid, octanoate, and pentadecanoate) and amino acids, in particular tryptophan and proline (Tables S1 and S2). This pattern of increased energy generation through fatty acid oxidation is a known response to endurance training.^37^ The increased uptake and mitochondrial transport of fatty acids suggests that difference in fatty acid oxidation between trained and UT individuals may be due to enhanced fatty acid transport into the mitochondria^38^ in trained individuals.

beta-Alanine is a non-proteinogenic amino acid, and is the limiting substrate in the biosynthesis of carnosine. Carnosine acts as buffer to stabilize pH in muscle and brain^39^ but has been shown to have antioxidant and antiglycating effects,^40^ thus potentially reducing the accumulation of advanced glycation end-products, toxic metabolic byproducts which have been implicated in several degenerative diseases, including aging.^41^ Increases in muscle carnosine content have been hypothesized to be an adaptation to long-term high-intensity training as demonstrated by higher plasma values found in bodybuilders^42^ and trained sprinters.^43^

Tryptophan metabolism and in particular the kynurenine pathway is emerging as a potential link between physical activity and health. The kynurenine pathway converts the amino acid tryptophan in NAD+, an important cofactor in energy metabolism. Interestingly, the intermediates of this pathway are also involved in inflammation, immune response, and neurotransmission,^44^ and have been associated with psychiatric conditions, such as depression^45^ and schizophrenia.^46^ Kynurenine metabolites accumulate with age and have recently been associated with bone loss, frailty, and increased mortality in older adults.^47^, ^48^, ^49^ Skeletal muscle tissue contributes to systemic kynurenine metabolism, and exercise training has been shown to affect the expression of kynurenine aminotransferase enzymes,^50^ possibly shifting kynurenine metabolism away from kynurenine, a toxic intermediate, toward the production of kynurenic acid.^51^

**Figure 9:**
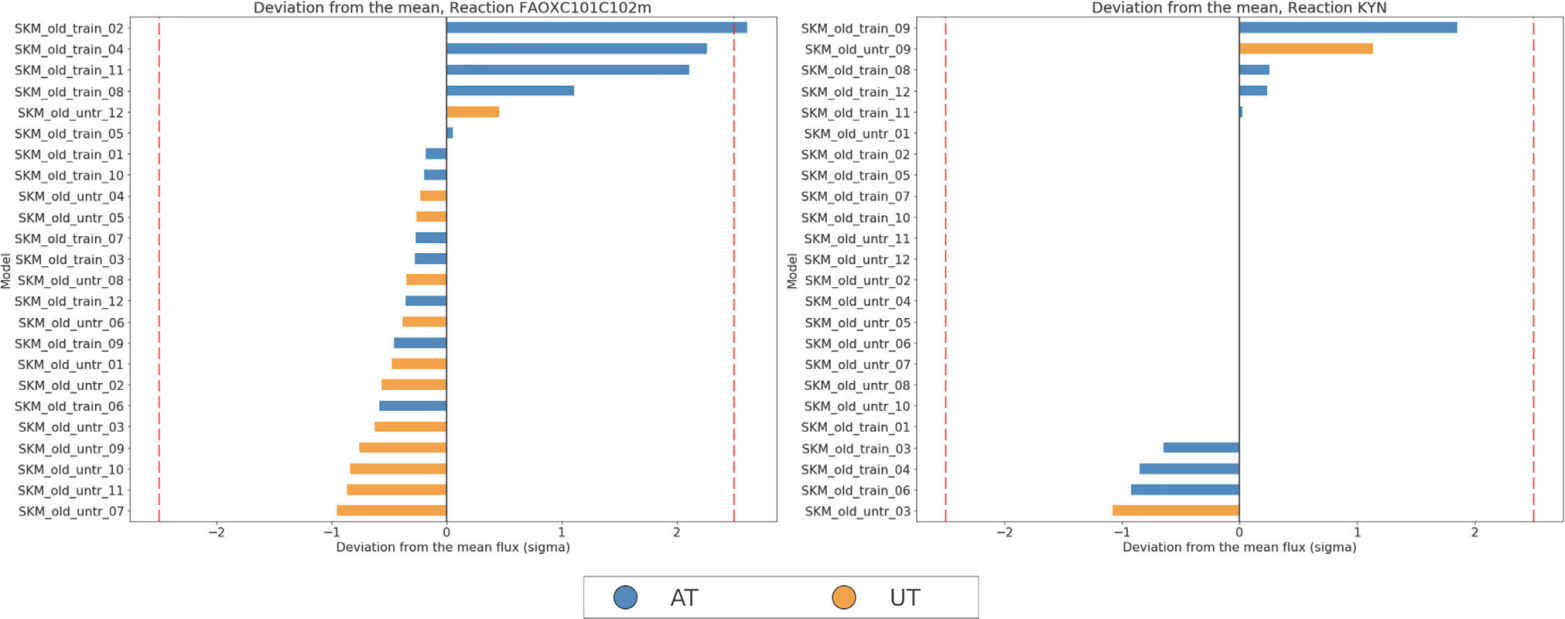
Plot of Normalized Fluxes for Selected Reactions Belonging to Significant Pathways Normalized fluxes for reactions belonging to some of the most represented pathway found to be associated with the intervention (resistance training). Left: fatty acid oxidation (mitochondrial), right: kynureninase (tryptophan metabolism). Dashed lines at ± 2.5 sigma signal a deviation of more than 95% from the mean.

**Figure 10:**
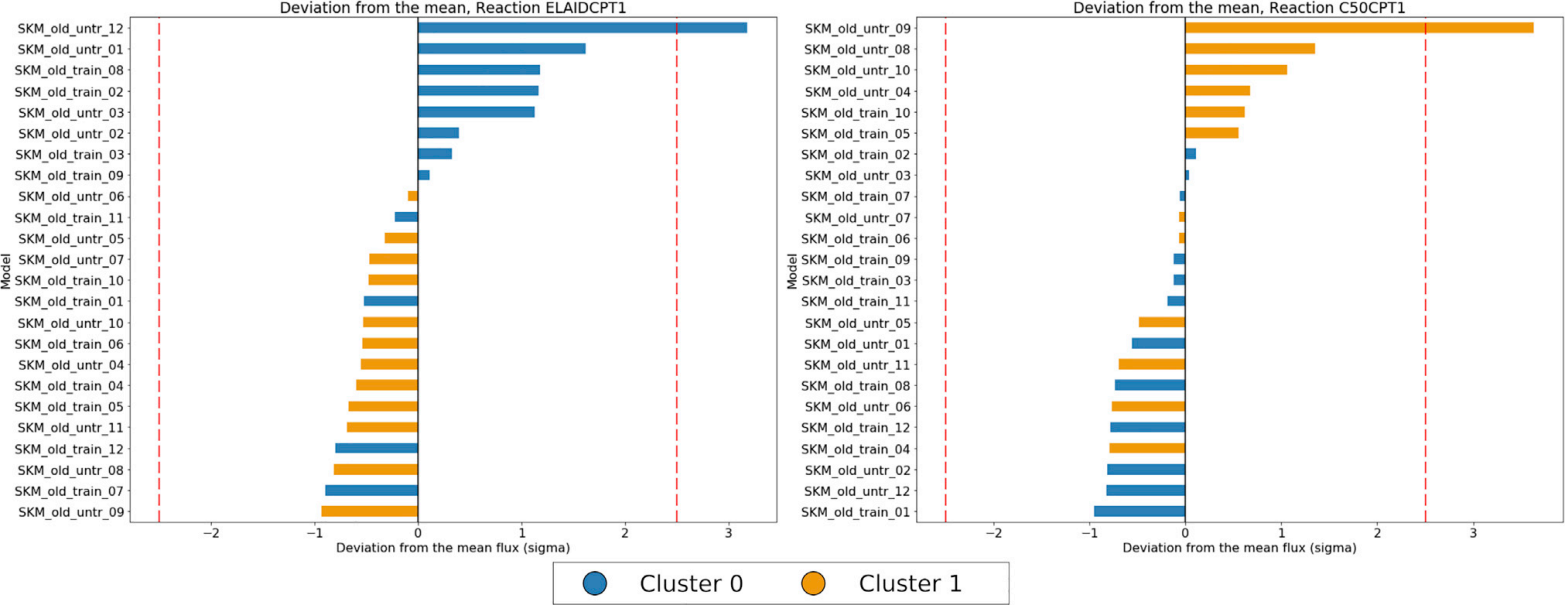
Plot of Normalized Fluxes for Selected Reactions Belonging to Significant Pathways Normalized fluxes for reactions belonging to some of the most represented pathway found to be associated with consensus clustering outcome. From left to right: carnitine palmitoyl transferase and butanoate mitochondrial transport (fatty acid oxidation). Dashed lines at ± 2.5 sigma signal a deviation of more than 95% from the mean.

Figures 9 and 10 show the normalized fluxes for significant reactions belonging to one of the selected pathways, for the treatment (Figure 9) and clustering (Figure 10) groups, respectively, and give an insight about individual responses, providing an insight about individual differences in the activity of the selected pathways. Figure 9 (right) shows the activity of kynureninase (normalized fluxes), a reaction responsible for kynurenine degradation, whose average flux is on average increased in AT models. This observation supports the conclusion that training has an effect on peripheral kynurenine metabolism, through upregulation of PGC-1α,^52^ reducing the accumulation of kynurenine and might explain the benefits of physical activity on mental health.

When comparing models of the two clusters, we can observe how distance is related to metabolic activity in certain pathways (Figure 10): For example, the flux through reaction carnitine palmitoyl transferase (reaction ID: ELAIDCPT1) is on average higher for the models in cluster 0 (Figure 10, right panel) while production of isovalerylcarnitine (reaction ID: C50CPT1) is on average higher in cluster 1. It is also important to notice how certain individuals have fluxes with a high deviation from the mean, more than 2.5 times the standard deviation. Even when considering flux degeneracy (i.e., many possible alternative flux distributions satisfy the same metabolic task), deviations of this magnitude are significant, and highlight the variability of the individual fluxes.

Our method was able to recover a biological signature of exercise training from a set of individualized GSMMs, identifying pathways associated with the metabolic intervention and enabled the characterization of individual metabolic heterogeneity in response to the same stimulus.

## Discussion

Patient-derived genome-scale models are a novel data class that integrates individual transcriptomics and other omics data with previous knowledge structured in large-scale metabolic networks. To quantify the degree of similarity between pairs of models in a large set, we investigated different distance metrics to express metabolic heterogeneity, a key step toward the application of ML algorithms to sets of GSMMs. Statistical learning methods have been successfully applied to find patterns in large amounts of data, without any previous knowledge about the system being examined, and have become relevant in the emerging field of personalized medicine in tasks, such as patient stratification,^53^^,^^54^ automated diagnosis of medical images,^55^ and planning of targeted therapeutic treatments.^56^ Still, despite its many advantages, ML has several shortcomings. Firstly, ML cannot offer mechanistic insights, just statistical correlations. Secondly, it is difficult to introduce in such algorithms any previous knowledge, for example, taken from the literature. Finally, ML models often trade explainability and transparency for predictive power.^57^ These are all seen as serious problems in the biomedical sciences, where knowing the mechanism of a disease or the rationale of a diagnosis is often preferable to pure predictive accuracy.

In this study we investigated the concept of distance between GSMMs, as a strategy to integrate previous knowledge about the metabolic network into ML algorithms. The structure of a GSMM simplifies the aggregation of different types of omics data in a system context: gene-level information (such as microarray or RNA sequencing), is organized and integrated, enabling the interpretation of the results in terms of the resulting system-level metabolic phenotype. The resulting individualized model is a snapshot of the status of the metabolic network of an individual in a particular condition that combines a metabolic knowledge base and experimental data. When ML algorithms are applied to these novel data structures and a pattern is detected, the relevant models can be further interrogated to generate and test hypotheses, going beyond mere correlations and toward mechanistic insights.

To harmonize these two approaches, we used distance between GSMMs as a proxy for metabolic similarity. Our initial hypothesis considered three different aspects of a GSMM, and assigned to each of them a different metric, to describe different but complementary components of metabolic distance. When we compared these three metrics—Jaccard distance, graph kernel similarity, and correlation between flux distributions—we found that the first two were very highly correlated, while the flux similarity metric had a medium to low correlation with the other two metrics. This observation made us reject the initial hypothesis in favor of a two-components interpretation of metabolic heterogeneity: a static component, which is the structure of the metabolic network, described by the Jaccard metric, and a dynamic one, which is the flux distribution.

Nevertheless, our proposed metric for flux distribution similarity, flux distribution correlation, was found to lack the resolution power to sufficiently separate the model classes and to be successfully used in ML applications. The problem of defining a suitable metric for flux similarity is still open. The solution space of the flux distribution is constrained by the topology of the metabolic network in combination with bounds set on particular reactions in the network. For example, lower and upper bounds on transport fluxes are used to incorporate information on medium composition and implement physiological conditions. We expect the metric based on flux correlation to be strongly dependent on these boundaries. Unknown variation and uncertainty in these boundaries can make the flux correlation metric less reliable. This could potentially explain why flux correlation performed worse than the other two metrics, when considering the separation of the classes, in both benchmark model sets.

Comparing the normalized fluxes between AT and UT models, we were able to extract a metabolic signature consistent with the effects of resistance training (Figure 8A). Fatty acid oxidation, kynurenine pathway, and beta-alanine pathway were the most represented pathways among the significant reactions identified. While the metabolic shift toward fatty acid oxidation is a known metabolic response to physical activity, this observation is a validation of our data-driven method to analyze sets of GSMMs parametrized with individual omics data. The identification of kynurenine and beta-alanine as pathways affected by endurance training is less trivial, and especially in the case of kynurenine pathway, establishes a mechanism to explain the beneficial effects of physical activity in older adults.

The results of the comparison between models of the two groups identified via consensus clustering (Figure 9B) shows that distance is indeed correlated with similarities in metabolic activity, also in large human models. Distance-based visualization of the model set in a metabolic health space also allowed to model the progress of a lifestyle intervention as trajectories. GSMMs are considered ideal platforms for the integration and interpretation of different types of high-throughput experimental data, but their scope is limited to modeling relationships between metabolic genes and the biochemical reactions that they encode. There is no description of biological processes, such as protein synthesis and gene regulation. Instead, gene expression and protein abundance data are used to constrain the model to a state which is closer to experimental observations by turning off reactions that are unsupported by data. The annotation of the model, specifically the link between gene, enzyme, and reaction levels, is thus fundamental for the process of “individualization,” i.e., the mapping of multi-omics data of a single individual to a “generic” human GSMM. We expect that the degree of heterogeneity exhibited by individualized models will increase as the annotation coverage of the generic human model's reactions increase. The inclusion of a particular reaction in an individualized model during the model-building phase, is conditioned by the expression of the genes associated with that reaction. The type of model-building algorithm, the threshold that defines when a gene is considered to be “expressed,” and the quality and coverage of the gene-protein reaction annotations of the “template” model, are all confounding factors that will influence whether a reaction will be included in a specific individualized model and consequently could alter the results of the analysis. Jaccard distance in particular is sensitive to these factors. Opdam et al.^58^ reviewed the effects of different combinations of model-building algorithms and parameter sets on the resulting model composition. Consensus analysis addresses this issue, reducing the sensitivity of the results to the influence of one particular metric. We also ensured to only compare models developed with the same algorithm and with the same sets of parameters (e.g., expression threshold). Nevertheless, increasing the standardization of the model-building process, and improving model annotation and coverage would increase the reliability of the results and the impact of this framework.

### Conclusions

In this study we investigated the concept of distance between patient-derived models comparing the properties of different distance metrics. We identified flux distribution and network topology/composition as the two complementary aspects of metabolic heterogeneity and developed a distance-based workflow that combines the ability of patient-derived metabolic models to provide previous knowledge of the structure of metabolic networks and a context for the integration and interpretation of omics data with the scalability and the pattern recognition capabilities of ML.

By applying this workflow to a set of patient-derived genome-scale models of aging muscle metabolism before and after a 12-week endurance training program, we identified subgroups of individuals expressing heterogeneous responses to the intervention. The fatty acids oxidation, kynurenine, and beta-alanine pathways were identified as differentially active between the two groups. Increased activity of these pathways in response to training explains the benefits of physical activity on metabolic and mental health during aging. We believe that the application of ML algorithms to sets of physiology-based computational models parametrized with individual molecular data will contribute significantly to the advancement of personalized medicine in the near future.

## Experimental Procedures

### Resource Availability

#### Lead Contact

Andrea Cabbia (a.cabbia@tue.nl)

#### Materials Availability

This study did not generate any new reagent or material.

#### Data and Code Availability

Patient-derived GSMMs of skeletal muscle models developed for this study are available and the custom codes to reproduce the results and figures are available at github.com/acabbia/tissueModels-distance. Gene expression data used in this study are available at Gene Expression Omnibus, under accession number GEO: GSE28422.

### Distance Metrics

#### Jaccard Distance

The Jaccard metric is used to compute the similarity score between pairs of binary vectors and has been previously applied to lists of reactions to compute the distance between pairs of models.^10^ The variability of these reconstructions was assessed computing the distance between the lists of reactions of every reconstruction pair using the Jaccard distance:

(Equation 1) J(A,B)=1−|A∩B|/|A∪B|.

It can be summarized as computing the “intersection over union” of two binary vectors to find their overlap. To allow for the analysis of models of different sizes, we summarized the reaction content of each model in a binary vector of length equal to the number of reaction in the template model (Recon2.2 in our case). For each reaction r in the parent model, if r is also included in the contextualized model, the rth position of the vector will be 1, else it will be 0.

#### Graph Kernels

Graph kernel functions were used to compute similarity scores between pairs of networks. Kernels are non-linear functions that map the data samples to a different dimensional space, known as the Hilbert space, usually with a higher dimensionality than the starting one. In this new space, the distance of samples represents their similarity. The exact coordinates in the Hilbert space, but rather their distance, is found by simply computing their inner products, an operation that is often computationally cheaper than the explicit computation of the new coordinates.

(Equation 2) K(X,Y)=φ(X)⋅φ(Y).

Kernel methods have been applied to heterogeneous types of data, such as biological sequence data,^59^ text,^60^ images,^61^ as well as graphs.^62^ Graph kernels are kernel functions that compute a similarity score between pairs of networks. We compared three different graph kernel functions: the random walk kernel,^63^ as implemented in Vishwanathan et al.,^64^ the Weisfeiler-Lehman subtree (WLS) kernel, proposed in Shervashidze et al.^65^, and the neighborhood subgraph pairwise distance kerne,l^66^ to compute a similarity score between pairs of genome-scale metabolic networks. Since our method is based on the analysis of hundreds of networks of several thousands nodes, we prioritized computational speed. We chose the last two kernels because their runtime complexity is lower than that of the random walk kernel, allowing them to be computed in a reasonable time. Figure S1 shows the runtime of the three kernels when applied to our library of 24 GSMMs of skeletal muscle metabolism. WLS has a runtime several orders of magnitude lower than the two alternatives and thus was chosen to be used in our pipeline. Highly connected metabolites, such as water, ATP, or NADH, also known as currency metabolites, were deleted from the models before computing the graph kernel similarity score.

#### Flux Correlation

Two main approaches can be considered to compute the similarity between pairs of constraint-based metabolic models, defined as: the first consists in comparing the functionality of two models, for example, comparing their ability to utilize different carbon sources or the minimal nutrient combinations predicted from the structure of the metabolic network.^67^ The similarity between the nutrient profiles of different models can then be compared by computing their Jaccard similarity (i.e., their overlap). We deemed this method to be unsuitable for the comparison of models of the same tissue type, since the differences in their carbon utilization profiles and minimal medium would be too small. Alternately, one could compare the functionality of two models by computing the overlap between sets of elementary flux modes (EFM).^68^ EFMs are minimal independent subnetworks of biochemical reactions that can operate at steady state and are considered the functional building blocks of metabolism. Enumerating and comparing sets of EFMs could give a measure of the functional similarity of the models; unfortunately the computation of EFMs becomes computationally too expensive for large networks. A second approach is to compute the distance between flux distribution vectors, obtained either via FBA or via Monte Carlo sampling of the model's solution space. The choice of metric, also in this case, is fundamental: the Euclidean metric is ineffective in high-dimensional spaces, composed of hundreds or thousands of dimensions, such as the reaction space of a GSMM (in such circumstances, as the number of dimensions tends to infinity, the Euclidean distance between any two points tends to converge). This effect is also known as the “curse of dimensionality.”^69^ In a study comparing a number of alternative metrics, Pearson's correlation and cosine similarity were found to have the best performances, in terms of both clustering accuracy and computational cost, when applied to high-dimensional microarray data.^70^ The choice of using FBA to obtain the flux distribution vector would be suboptimal for several reasons: first, FBA results are scarcely reproducible, mainly due to the degeneracy of stoichiometric networks (many different flux distributions can satisfy the same objective function) and to its sensitivity to the particular software or algorithm used to solve the linear programming problem. In addition, FBA imposes the use of an arbitrary optimization objective, meaning that each flux distribution found with FBA is inherently biased, and not representative of the complexity of the entire solution space of a constraint-based model. Possible alternatives, such as geometric FBA,^71^ so far remain hampered by excessive computational requirements, which limit their application in large model sets that are the focus of this analysis. As a measure of similarity between flux distributions, we will take instead the absolute Pearson's correlation coefficient between pairs of normalized flux vectors. These vectors were obtained by averaging 1,000 random samples of the solution space of a metabolic model, and were normalized by subtracting the mean flux value of all individuals and dividing them by their standard deviation. Using random sampling instead of FBA reduces the bias of the analysis, since it is not necessary to define an objective function.

### Metrics Comparison

We hypothesized that, since each of these three different aspects of a GSMM encapsulates different aspects of metabolic heterogeneity, they would need different definitions of distance. Two recently published large model sets of microbial and human cancer metabolism, the AGORA model set,^10^ which includes 818 metabolic reconstructions of human gut bacteria, and a subset of the PD-GSMM set, which includes hundreds of cancer patient-derived tissue-specific models,^9^ were used were used to test this hypothesis. For each of these two model sets, we computed the distance between every pair of models using three different distance metrics. The inverse of the flux correlation and graph kernel similarity scores were used as metrics of distance to allow the comparison between the three metrics. The resulting (symmetric) pairwise distance matrices were clustered with hierarchical clustering (average linkage) and presented as heatmaps (Figure 1). To test whether the three metrics actually are complementary and describe different aspects of metabolic heterogeneity, we used the Mantel test^72^ to compute the correlation between distance matrices, since the assumptions of independence, normality, and homoscedasticity do not hold for metabolic flux distirbutions.

### Data

The workflow described in the section above was applied to a set of metabolic models that was developed for this study. This model set describes the skeletal muscle metabolism of 12 older individuals, and was created from longitudinal gene expression data, collected before and after a resistance training program of 12 weeks. The gene expression dataset from which the skeletal muscle metabolism models were developed was collected during a study on the acute and long-term effects of resistance training on skeletal muscle gene expression in older adults^34^ and has been published on the Gene Expression Omnibus^73^ under accession number GEO: GSE28422. Gene expression data were measured with microarray technology (platform HG U133 Plus 2.0). Older participants (n = 12, 84 ± 1 years old) included 6 men and 6 women. All subjects participated in 12 weeks of progressive resistance training consisting of bilateral knee extensions with 3 × 10 reps at 70% of 1-RM, and 3 days/week for a total of 36 training sessions. Vastus lateralis biopsies were obtained in conjunction with the first and last training session. We refer to the former as UT status, and to the latter as AT status. From this dataset we developed 24 patient-derived GSMMs, two for each subject, respectively, for the UT and AT status.

### Model Building

Several different methods for the integration of multi-omics data in GSMMs have been reviewed in Opdam et al.^58^ The dataset introduced in the section above was used, in combination with a template human metabolic network reconstruction, Recon2.2,^33^ to create a collection of patient-derived models of older adults' skeletal muscle metabolism in UT and trained conditions. Gene expression data are used to identify model reactions with high experimental support. To convert gene expression data into a confidence score for Recon2.2 reactions, microarray probeset intensity value was log transformed and sorted by decreasing expression values. When two or more probes mapped to the same gene, we merged them averaging their expression value. The top 30% highly expressed probes were assumed to be “high confidence.” Using this gene-level confidence score and the gene-protein reaction rules included in the Recon2.2 model, we computed a reaction-level confidence score, which was then used as input for the model-building algorithm. The CORDA algorithm 8 generates a draft model, including all the high-confidence reactions and a minimal amount of lower-confidence reactions, such that the resulting network is fully connected and that all the reactions can carry flux. After the models had been drafted by the algorithm, they underwent substantial manual curation and validation, starting with a comparison with known physiological properties of the tissue being examined. This resulted in the manual addition of reactions that were excluded by the algorithm but are known to be active in myocytes (e.g., ATP synthase). The models completed muscle-specific functional tests, such as ATP production from a range of different sources and glycogen synthesis. The outcome of this process was a collection of 24 PD-GSMMs of muscle metabolism in older individuals (n = 12 AT, n = 12 UT).

### Other Software

We used the CobraPy^74^ library to work with GSMMs and for FBA. The patient-derived metabolic modes were created with the CORDA algorithm 8; the GraKeL^75^ python library was used for the graph kernels computations and the SciKit-Learn^76^ for hierarchical clustering. We used METIS^77^ to perform consensus clustering analysis. All the computations were performed in Python 3.6 on a Ubuntu 18.04 machine with Xeon CPU E5-1620 at 3.50 GHz and 16 GB RAM.

## Supplemental Information

Document S1. Figure S1

Table S1. List of Reactions with Significantly Different Activity in Cluster 0 versus Cluster and Associated p Values These data are relative to the Kruskal-Wallis test after comparing models belonging to the two clusters.

Table S2. List of Reactions with Significantly Different Activity in AT versus UT Models and Associated p Values These data are relative to the Kruskal-Wallis test after comparing models belonging to the two treatment groups (i.e., trained/untrained).

Document S2. Article plus Supplemental Information
