# A Distance-Based Framework for the Characterization of Metabolic Heterogeneity in Large Sets of Genome-Scale Metabolic Models

**DOI:** 10.1016/j.patter.2020.100174

**Published:** 2020-12-11

**Authors:** Andrea Cabbia, Peter A.J. Hilbers, Natal A.W. van Rie

## Main Text

(Patterns *1*, 100080-1–100080-16; September 11, 2020)

In the originally published version of this article, the graphs in Figure 2 do not contain the explained variance with the axes. The corrected Figure 2 is shown here.

Also, the legend for Figure 3 was mistakenly used for Figure 7 as well. The correct legend for Figure 7 is as follows: Consensus clustering results are shown as colored shapes. Cluster 0 (orange) includes mostly untrained models; cluster 1 (blue) is composed mostly of trained models. Four individuals (7, 8, 9, and 11) show the largest shift during the training intervention, starting from the orange cluster and shifting toward other AT models in the blue cluster after the end of the treatment.

Finally, the originally published version mentions both “endurance training” and “resistance training,” whereas in the original study that collected the data (Raue et al., 2012, J Appl Physiol *112*, 1625–1636, https://doi.org/10.1152/japplphysiol.00435.2011), the authors mention resistance training. Because of this, all instances of “endurance training” in the article have been replaced by the term “resistance training.”

The article has now been corrected online, and the authors apologize for any confusion these errors may have caused.

**Figure:**
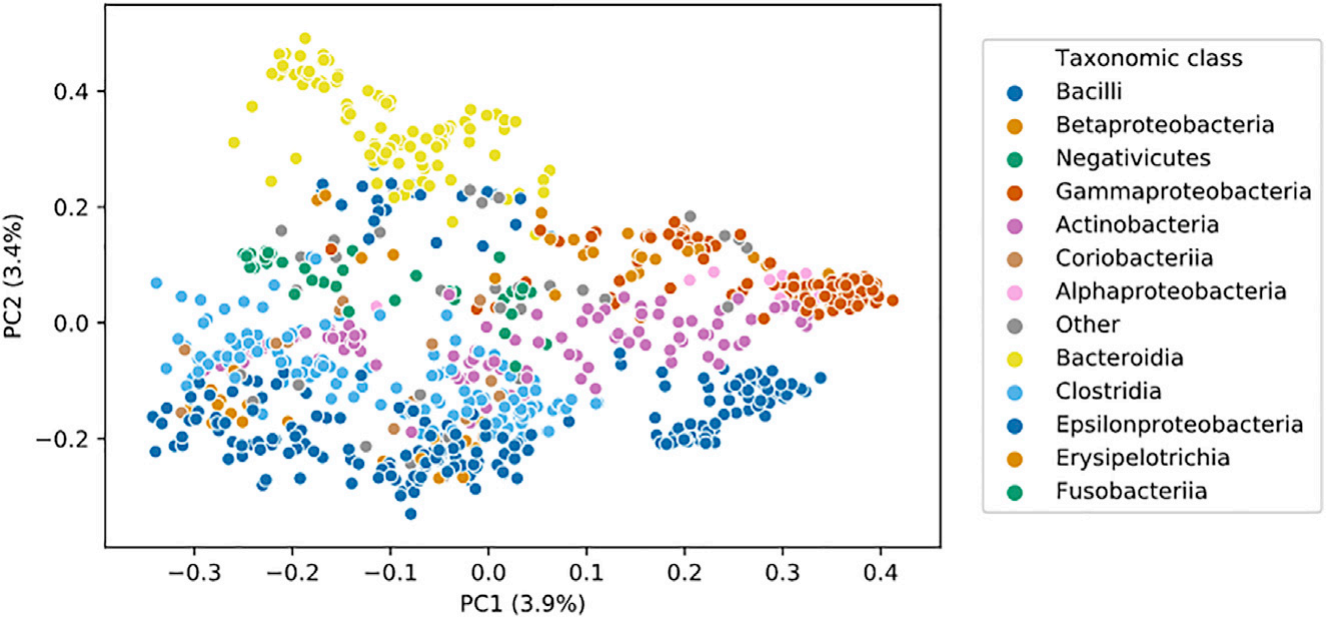
Figure 2. Visualization of the Full AGORA Microbial Model Set (corrected)

**Figure:**
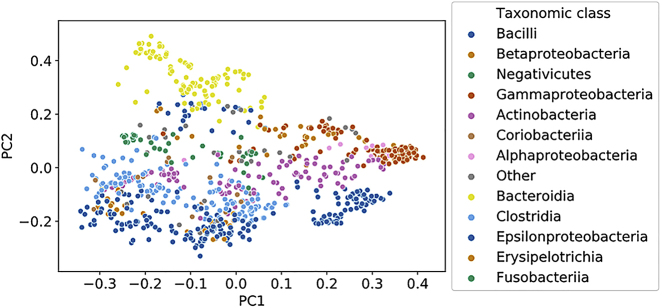
Figure 2. Visualization of the Full AGORA Microbial Model Set (original)

