# Supplementary material for: A Distance-Based Framework for the Characterization of Metabolic Heterogeneity in Large Sets of Genome-Scale Metabolic Models
Source: Patterns (N Y). 2020 Aug 6;1(6):100080. doi: 10.1016/j.patter.2020.100080 (PMC7660451; doi:10.1016/j.patter.2020.100080)
Supplement: Document S1. Figure S1 [file mmc1.pdf]

**PATTER, Volume *1***

## Supplemental Information

# A Distance-Based Framework for the Characterization of Metabolic Heterogeneity in Large Sets of Genome-Scale Metabolic Models

**Andrea Cabbia, Peter A.J. Hilbers, and Natal A.W. van Riel**

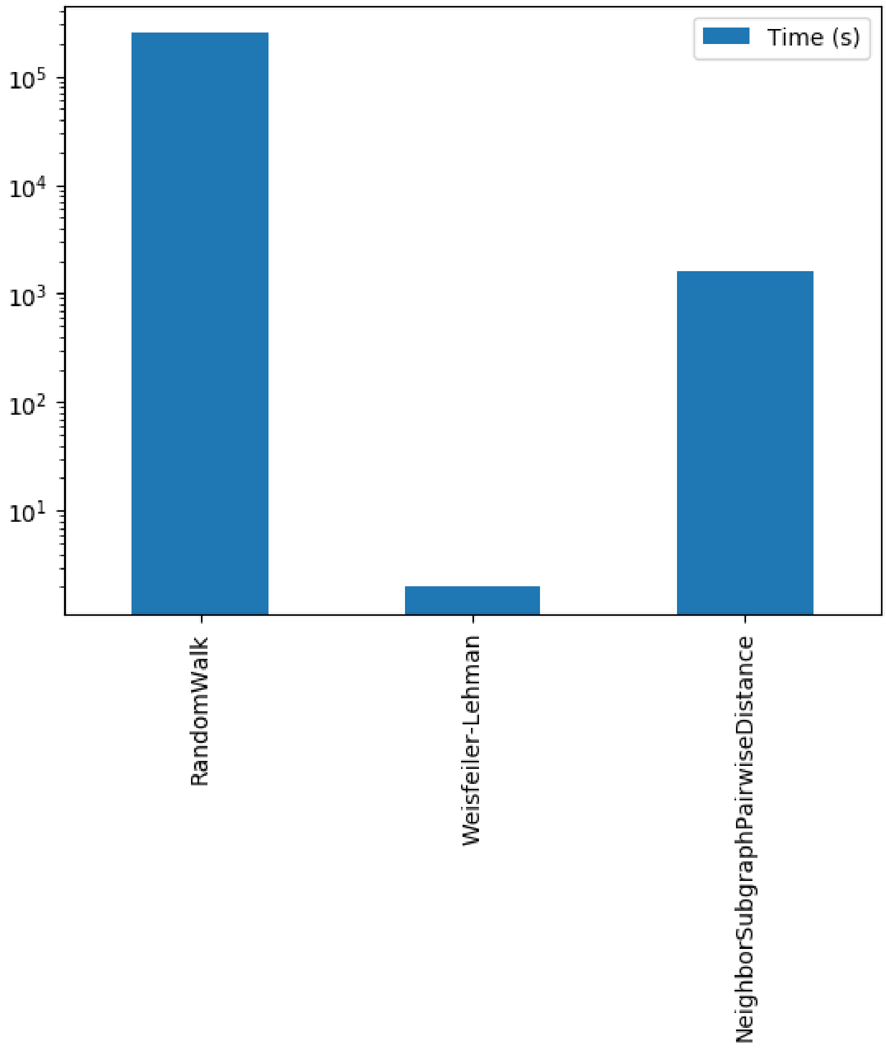


Figure 1: **Comparison between runtimes for three different Graph kernels**
We compared the performances of three graph kernel algorithms (Random walk, Weisfeiler-Lehman, Neighborhood Subgraph Pairwise Distance), when computing pairwise distances between 24 patient-derived human models of skeletal muscle metabolism. The content of and size of the models is described in the main text. The Weisfeiler-Lehman kernel is several order of magnitudes faster then the other methods when used to compute similarity between large human genome-scale metabolic models.
